# What Multiple Myeloma With t(11;14) Should Be Classified Into in Novel Agent Era: Standard or Intermediate Risk?

**DOI:** 10.3389/fonc.2020.538126

**Published:** 2020-10-26

**Authors:** Wen Gao, Juan Du, Junru Liu, Huixing Zhou, Zhiyao Zhang, Yuan Jian, Guangzhong Yang, Guorong Wang, Ying Tian, Yanchen Li, Yin Wu, Weijun Fu, Juan Li, Wenming Chen

**Affiliations:** ^1^ Department of Hematology, Myeloma Research Center of Beijing, Beijing Chao-Yang Hospital, Capital Medical University, Beijing, China; ^2^ Department of Hematology, The Myeloma & Lymphoma Center, Changzheng Hospital, The Second Military Medical University, Shanghai, China; ^3^ Department of Hematology, The First Affiliated Hospital of Sun Yat-sen University, Guangzhou, China

**Keywords:** autologous stem cell transplantation, multiple myeloma, novel agent, 1q21 gain, t(11;14)

## Abstract

**Objective:**

To investigate the prognostic value of t(11;14) for *de novo* multiple myeloma (MM) patients in novel agent era.

**Methods:**

A total of 455 patients with fluorescence *in situ* hybridization (FISH), before treatments from three hospitals in China, were included in the study. All patients received autologous stem cell transplantation (ASCT) after induction therapy as consolidation. High risk (HR) cytogenetics were defined as t(4;14), t(14;16), and/or del 17p.

**Results:**

A total of 152 patients were in the HR group. Of patients without HR cytogenetics, 55 were in the t(11;14) group, and 248 were in the standard risk (SR) group without t(11;14). Gain in 1q21 was observed in 38.9% patients with t(11;14). There were no differences in median progression free survival (PFS) and overall survival (OS), respectively, between patients in the t(11;14) group and those in the SR group. Patients in the t(11;14) group had the longer median PFS and OS, respectively, compared with those in the HR group. Regardless of coexisting with 1q21 gain or not, patients in the t(11;14) group still had similar median PFS and OS compared to those in the SR group. Finally, multivariate analysis indicated that including 1q21 gain and bone marrow plasma cell with CD20 expression, no variables were found to predict the outcome of the t(11;14) group in our cohort.

**Conclusions:**

These results confirm that outcomes of t(11;14) MM are similar to standard risk patients when they receive novel agent induction therapy consolidated by ASCT. Gain of 1q21 coexists with t(11;14) frequently. In addition, both bone marrow plasma cell with CD20 expression and 1q21 gain have no impact on median PFS or OS for patients with t(11;14).

## Introduction

Multiple myeloma (MM) is still incurable. But overall survival for these patients has been improved significantly with novel agents in combination with autologous stem cell transplantation (ASCT). With the rapid changes of treatment pattern, the role of prognostic factors for MM has to be reevaluated accordingly. Among MM patients, about 16–24% can be detected with translocation t(11;14)(q13;q32), which ranks with the most common chromosomal translocation (1–5). Two decades ago, with or without consolidation by ASCT, MM patients with t(11;14) were classified into standard risk, which was supported by some studies (1, 3, 6, 7). In a novel agents era, there is controversy regarding the prognosis with t(11;14). The research from Mayo Clinic showed that overall survival (OS) for t(11;14) MM was shorter than that for standard risk patients, and this was still the case even with early ASCT (8, 9). On the contrary, other studies suggested that MM with t(11;14) had a similar prognosis to the standard risk group (10–12). Although the widely used Revised International Staging System (R-ISS) did not take t(11;14) into account for staging (13), more and more people begin to consider that multiple myeloma with t(11;14) should be classified into a novel agent era: standard or intermediate risk?

In this context, we retrospectively reviewed patients from three Chinese hospitals to assess the survival outcomes of 455 MM patients under treatment of novel agents in combination with ASCT. Among them, 55 patients were detected with t(11;14).

## Methods

### Patients

Between March 2003 and January 2018, 455 patients with newly diagnosed symptomatic myeloma had received fluorescence *in situ* hybridization (FISH) prior to the treatment, and then they received at least one ASCT after induction therapy at Beijing Chaoyang Hospital, Shanghai Changzheng Hospital, and Guangzhou Zhongshan Hospital. Upfront ASCT has been recommended to all eligible MM patients with or without FISH abnormalities under our routine practice. All patients received ASCT within 12 months of starting treatment for MM. Eight patients received second ASCT as salvage therapy after relapse. Patients who coexisted with amyloidosis at diagnosis were excluded. To evaluate the impact of t(11;14) on survival, we further divided the patients into those with t(11;14) and without t(4;14), t(14;16), and del 17p [t(11;14) group) (n=55)], those without t(11;14), t(4;14), t(14;16), and del 17p [standard risk (SR) group] (n=248), and those with t(4;14), t(14;16), and/or del 17p [high risk (HR) group] (n=152). Follow-up data were obtained until January 2019, and the median of the follow-up period was 35.8 (range 6–119) months. The study was approved by the Beijing Chaoyang Hospital Institutional Review Board and conducted in accordance with the declaration of Helsinki.

Among the patients enrolled in this study, some had already participated in clinical trials under local hospital routine practice. Data were obtained from the three hospital databases, respectively, which were created and maintained prospectively. All MM diagnosis and treatment responses were based on the International Myeloma Working Group (IMWG) criteria (14, 15).

FISH screening was performed using DNA probes (Abbott Molecular) targeting at least one of the following chromosomal abnormalities: 17p13 deletion, t(11;14), t(4;14), t(14;16), and 1q21 gain. A total of 200 interphase nuclei were analyzed. The cutoff values were as follows: 20% for 17p13 deletion and 1q21 gain; 10% for t(11;14), t(4;14), and t(14;16) (16). Among 455 patients, 37 were not purified but directly analyzed as bone marrow mononuclear cells.

### Statistical Analysis

We summarized categorical variables as proportions and continuous variables as median (range). X^2^-test was used to compare categorical variables among different groups with Fisher’s exact test when appropriate. Non-parametric Kruskal–Wallis test was used to compare continuous variables. Progression free survival (PFS) was defined as the duration from initiation of therapy to first evidence of disease progression or death. Patients without evidence of progressive disease were censored at the date of last follow-up. OS was calculated from the date of treatment initiation until the date of death or date of last follow-up. PFS and OS were estimated with the method of Kaplan and Meier and compared among groups using log rank test. For multivariate analysis, factors associated with PFS and OS were introduced into a Cox proportional hazards model.

To identify predictors of outcome for patients in the t(11;14) group, univariate analysis was performed with age ≧̸50 vs. <50 years; hemoglobin ≧̸10 vs. <10 g/dl; bone marrow plasma cell (BMPC)≧̸30% vs. <30%; bone marrow plasma cell with vs. without CD20 expression and with vs. without 1q gain; light chain myeloma vs. others; ISS (International Staging System) I vs. II/III stage; less than vs. at least VGPR before ASCT, and less than vs. at least VGPR at three months after ASCT as independent variables to confirm their association with median PFS and OS. Variables with a P-value <0.166 (For PFS) or P-value <0.4 (For OS) on univariate analysis and bone marrow plasma cell with vs. without CD20 expression were analyzed as predictor variables in multivariate Cox proportional hazards model.

IBM SPSS v21 software (SPSS Inc., Chicago, IL, USA) was used for statistical analysis.

## Results

### Patients Baseline Characteristics

The baseline characteristics of the three groups are summarized in **Table 1**. Among 455 patients, 65 patients were found with t(11;14) alone or t(11;14) plus additional chromosome abnormality, and 55 patients were included in the t(11;14) group (without t[4;14], t[14;16], and del 17p). A gain of 1q21 was detected in 39.7% of 446 patients and in 38.9% of 54 patients with t(11;14). Both gender and median age at diagnosis were similar across the groups. For the M-protein isotype, light chain was more prevalent in the t(11;14) group (36.3%, p<.001). A similar tendency was observed for IgD in the t(11;14) group (9.1%). Proportion of patients with international staging system (ISS) III stage was similar across the groups. There were no differences in laboratory tests, induction therapy, and conditioning regimens among three groups respectively. The cytogenetic patterns of the three groups are exhibited in **Table 2**.

**Table 1:** The Baseline Clinical Characteristics of the Study Populations at Diagnosis (n=455).

Characteristic	All patients	t(11;14) group	Standard risk	High risk	P
	(n=455)	55	248	152	
Male, n(%)	272 (59.8)	35 (63.6)	146 (58.9)	91 (59.9)	0.823
Age, y(median/range)	53 (23-69)	50 (30-65)	54 (23-66)	53 (25-69)	0.062
M-protein isotype					
IgG, n (%)	240 (52.7)	22 (40)	128 (51.6)	90 (59.2)	0.044
IgA, n (%)	94 (20.7)	5 (9.1)	55 (22.2)	34 (22.4)	0.066
IgD, n (%)	18 (4.0)	5 (9.1)	7 (2.8)	6 (3.9)	0.098
Light chain, n (%)	89 (19.6)	20 (36.3)	51 (21.4)	18 (11.9)	<0.001
Others, n (%)	14 (3.1)	4 (7.3)	6 (2.4)	4 (2.6)	0.150
ISS I/II, n (%)	288 (63.3)	41 (74.5)	152 (61.3)	95 (62.5)	0.175
ISS-III, n (%)	167 (36.7)	14 (25.5)	95 (38.3)	57 (37.5)	0.190
LDH, median (range) (n=413)	161 (67-732)	162 (82-704)	161 (84-615)	160 (67-732)	0.63
Hb, g/L, median (range)	97 (44-159)	105 (57-159)	95 (44-151)	94.5 (51-150)	0.111
Platelets, 10^9^/L, median (range)	196 (28-485)	200 (85-325)	205 (31-841)	178 (28-386)	0.032
Creatine, umol/L, median (range)	77.9 (30-881)	78.3 (36.3-404)	78 (31-777)	76 (30-881)	0.997
Calcium, mmol/L, median (range)	2.38 (1.65-5.08)	2.46 (2.03-4.29)	2.35 (1.8-5.09)	2.35 (1.65-4.11)	0.082
BMPC, %, median (range)	31 (0.5-96.5)	35 (10-89)	29.3 (1-96.5)	32.5 (0.5-94)	0.090
Novel agents containing regimens during induction, n (%)					
PI based, n (%)	403 (88.6)	50 (90.9)	221 (89.1)	132(86.8)	0.708
IMiDs based, n (%)	2 (0.4)	0	2 (0.8)	0	
PI + IMiDs based, n (%)	5 (1.1)	0	3 (1.2)	2 (1.3)	
Conventional therapy, n(%)	45 (9.9)	5 (9.1)	22 (8.9)	18 (11.8)	0.640
Conditioning regimens					
Melphalan alone, n(%)	224 (49.2)	30 (54.5)	126 (50.8)	68 (44.7)	0.125
Other regimens, n(%)	231 (50.8)	25 (45.5)	122 (49.2)	81 (55.3)	0.125

t(11;14) group, defined as with t(11;14) and without t(4;14), t(14;16), and del 17p; Standard risk, defined as the absence of del17p, t(4;14), t(14;16), and t(11;14); High risk, defined as the presence of any of del17p, t(4;14), and/or t(14;16). P-value for Kruskal-Wallis test for continuous variables and Fisher’s exact test for categorical variables.

ISS, International Staging System; LDH, lactate dehydrogenase; Hb, Hemoglobin; BMPC, Bone Marrow Plasma Cell; PI, Proteasome inhibitors; IMiDs, Immunomodulatory drugs.

**Table 2:** Cytogenetic Proﬁles of Patients Based on Interphase Fluorescent in Situ Hybridization (n=455)^a^.

Cytogenetic abnormality	t(11;14) group	Standard risk	High risk	p
	(n=55)	(n=248)	(n=152)	
t(11;14), n (%)	55 (100)	0	10 (6.6)	
t(4;14), n (%)	0	0	84 (55.3)	
t(14;16), n (%)	0	0	15 (9.9)	
Del 17p, n (%)	0	0	70 (46.1)	
1q21 gain, n (%)	21 (38.9)^b^	80 (32.8)	76 (51.4)	0.001

a t (11;14) group, defined as with t(11;14) and without t(4;14), t(14;16), and del 17p; Standard risk, defined as the absence of del17p, t(4;14), t(14;16), and t(11;14); High risk, defined as the presence of any of del17p, t(4;14), and/or t(14;16).

b Among the t(11;14) group, one patient had missing data for 1q21 gain; among the standard risk group, 4 patients had missing data for 1q21 gain; among the high risk group, 4 patients had missing data for 1q21 gain.

### Induction Therapy, Conditioning Regimens, and Maintenance Therapy

The induction regimens for the three groups of patients are shown in **Table 1**. About 90% of patients received proteasome inhibitors (PI)-based induction, whereas the other 10% of patients received conventional therapy. In addition, a small proportion of patients (about 1.5%) received combinations containing lenalidomide with or without PI. Between the three groups, it was similar for the percentage of patients receiving different classes of induction therapies. As a conditioning regimen, 200 mg/m^2^ melphalan was used for nearly 50% of patients, and a small proportion of patients received melphalan in combination with Bortezomib or total body irradiation. Nearly 50% of patients received busulfan and cyclophosphamide in combination with etoposide as conditioning regimen, and a small proportion of patients received busulfan in combination with cyclophosphamide. Between the three groups, it was still similar for the percentage of patients receiving different classes of conditioning regimens (**Supplementary Table 1**). Of the 455 patients, 311 received conventional agents as maintenance, 97 patients received maintenance with novel agents, and the other 47 patients received no maintenance. Between the three groups, there were no differences for the percentage of patients receiving different classes of maintenance (**Supplementary Table 2**).

### Response to Induction or ASCT

Response to induction was assessed in the t(11;14) group, the SR group, and the HR group, respectively. The proportion of patients with stringent complete response (sCR), complete response (CR), very good partial response (VGPR), at least VGPR, partial response (PR), minimal response (MR), stable disease (SD), and progressive disease (PD) as best response to induction or ASCT are shown in **Table 3**. The differences were observed in the proportion of patients who achieved at least VGPR before ASCT in the three groups (56.4% vs 73.8% vs 76.9% and P=.014). The lowest at least VGPR was in the t(11;14) group. After ASCT, no differences were observed in the percentage of patients who achieved at least VGPR in the three groups (65.5% vs 79% vs 76.9% and P=.069) (**Table 3**). It makes sense to assume that t(11;14) MM may benefit more from ASCT for improvement of response compared with the other two groups. Considering the heterogeneity of conditioning regimens, it is necessary to make clear which is the better containing regimen for t(11;14) MM: busulfan containing regimens or melphalan containing regimens? Further analysis did not confirm that busulfan containing regimens did better than melphalan containing regimens in terms of at least VGPR after ASCT (**Supplementary Table 3**).

**Table 3:** The Response of the Study Populations at Diagnosis (n=455).

Characteristic	All patients	t(11;14) group	Standard risk	High risk	P
	(n=455)	55	248	152	
Pre-ASCT response, n (%)					
sCR	37 (8.1)	4 (7.3)	24 (9.7)	9 (5.9)	0.431
CR	111 (24.4)	11 (20)	64 (25.8)	36 (23.7)	0.692
VGPR	183 (40.2)	16 (29.1)	95 (38.3)	72 (47.4)	0.042
At least VGPR	331 (72.7)	31 (56.4)	183 (73.8)	117 (76.9)	0.014
PR	96 (21.1)	15 (27.3)	52 (21)	29 (19.1)	0.430
MR	11 (2.4)	3 (5.5)	6 (2.4)	2 (1.3)	0.211
SD	10 (2.2)	6 (10.9)	4 (1.6)	0	
PD	4 (0.9)	0	2 (0.8)	2 (1.3)	
Unknown	3 (0.7)	0	1 (0.4)	2 (1.3)	
The response at three months after ASCT, n (%)					
sCR	53 (11.6)	3 (5.5)	34 (13.7)	16 (10.5)	0.21
CR	156 (34.3)	17 (30.9)	93 (37.5)	46 (30.3)	0.299
VGPR	133 (29.3)	16 (29.1)	69 (27.8)	48 (31.5)	0.712
At least VGPR	342 (75.2)	36 (65.5)	196 (79)	110 (76.9)	0.069
PR	58 (12.7)	11 (20)	28 (11.3)	19 (12.5)	0.214
MR	7 (1.5)	1 (1.8)	6 (2.4)	0	
SD	5 (1.1)	3 (5.5)	1 (0.4)	1 (0.7)	
PD	10 (2.2)	1 (1.8)	6 (2.4)	3 (2.0)	
Unknown	33 (7.3)	3 (5.5)	11 (4.4)	19 (12.5)	0.012

t(11;14) group, defined as with t(11;14) and without t(4;14), t(14;16), and del 17p; Standard risk, defined as the absence of del17p, t(4;14), t(14;16), and t(11;14); High risk, defined as the presence of any of del17p, t(4;14), and/or t(14;16).

ASCT, Autologous stem cell transplantation; sCR, stringent complete response; CR, complete response; VGPR, very good partial response; PR, partial response; MR, minimum response; SD, stable disease; PD, progression of disease.

### Survival Outcomes

**Figure 1:**
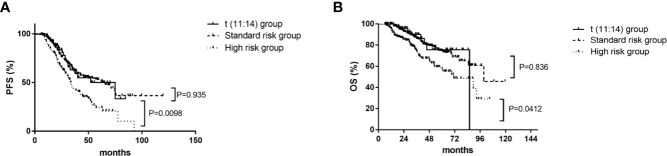
Impact on PFS and OS with t(11;14) group (n=55), standard risk group (n=248), and high risk group (n=152). **(A)** Impact on PFS with t(11;14) group, standard risk group, and high risk group. **(B)** Impact on OS with t(11;14) group, standard risk group, and high risk group. PFS, progression free survival; OS, overall survival; t(11;14) group, defined as with t(11;14) and without t(4;14), t(14;16), and del 17p; Standard risk, defined as the absence of del17p, t(4;14), t(14;16), and t(11;14); High risk, defined as the presence of any of del17p, t(4;14), and/or t(14;16).

**Figure 2:**
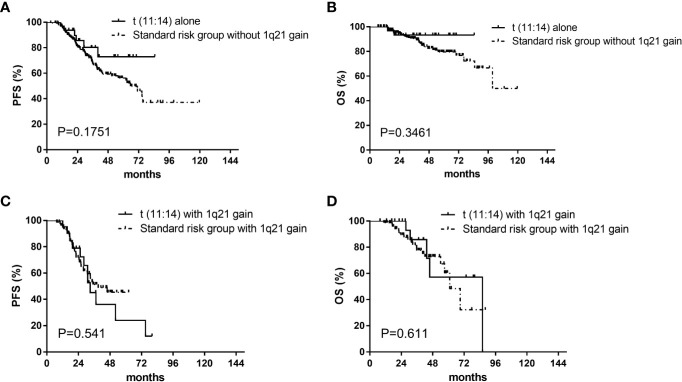
Impact on PFS and OS with t(11;14) group and standard risk group without or with 1q21 gain. **(A)** Impact on PFS with t(11;14) alone (n=33) and standard risk group (n=164) without 1q21 gain. **(B)** Impact on OS with t(11;14) alone (n=33) and standard risk group (n=164) without 1q21 gain. **(C)** Impact on PFS with t(11;14) (n=21) and standard risk group (n=80) with 1q21 gain. **(D)** Impact on OS with t(11;14) (n=21) and standard risk group (n=80) with 1q21 gain. PFS, progression free survival; OS, overall survival; t(11;14) group, defined as with t(11;14) and without t(4;14), t(14;16), and del 17p; Standard risk, defined as the absence of del17p, t(4;14), t(14;16), and t(11;14).

There were no differences in median progression free survival (PFS) (52 [95% CI, 26–78] vs 63 [95% CI, 44–82] months, P=.935) and overall survival (OS) (86 vs 100 months, P=.836) respectively between patients in the t(11;14) group (n=55) and those in the SR group (n=248) (**Figure 1**). The median PFS were 52 (95% CI, 26–78) and 33 (95% CI, 30–36) months for patients in the t(11;14) group (n=55) and those in the HR group (n=152) respectively (P=.009). There was a similar trend toward longer median OS for the t(11;14) group compared with HR group [median OS of 86 (no CI) vs 71 (95% CI, 48–94) months, P=.041] (**Figure 1**). In addition, we compared the outcome between the t(11:14) group and the SR group excluding patients with 1q21 gain. No differences were found in both median PFS[not reach vs 65.5 (95% CI, 51.4–79.6) months, P=.175]and OS (not reach vs 99.5 months, P=.346) between t(11;14) alone (n=33) and SR group (n=164) (**Figures 2A, B**). It suggested that even without 1q21 gain, patients with t(11:14) alone still had comparable outcome against those with SR. Finally, we further analyzed the impact of 1q21 gain on the outcome comparison between t(11;14) group and SR group. The similar survival tendency was found between t(11;14) alone plus 1q21 gain (n=21) and SR plus 1q21 gain (n=80). No differences were found in both median PFS[33 (95% CI, 26.1–39.9) vs 39.1 months, P=.0.541] and OS [86 vs 61 months (95% CI, 49.9–72.0), P=.611] between t(11;14) alone (n=21) and SR group (n=80) including 1q21 gain (**Figures 2C, D**). It suggested that regardless of without or with 1q21 gain, patients in the t(11:14) group had comparable outcome against those in the SR group.

### Predictors of Outcome for Patients in the t(11;14) Group

The results of analysis showed that there were no variables to predict median OS. The presence of 1q gain was associated with reduced median PFS in univariate but not in multivariate analysis (**Tables 4**, **Table 5**).

**Table 4:** Effect of baseline characteristics and response on PFS in the t(11;14) group (n=55).

Variables	Univariate analysis			Multivariate analysis		
	HR	95%CI	p	HR	95%CI	p
Age ≧̸50 vs <50 years (30 vs 25)	1.952	0.767-4.970	0.161	0.635	0.115-3.507	0.602
Hb ≧̸10 vs <10 g/dl (34 vs 21)	0.946	0.372-2.405	0.907			
BMPC ≧̸30% vs <30% (29 vs 26)	0.345	0.122-0.979	0.045	0.206	0.024-1.759	0.149
BMPC with vs without CD20 expression (18 vs37)	0.467	0.105-2.072	0.316	2.652	0.122-5.782	0.534
with vs without 1q gain (21 vs 33)	0.297	0.110-0.803	0.017	0.274	0.030-2.482	0.250
Light chain myeloma vs others (20 vs 35)	0.990	0.374-2.619	0.984			
ISS I vs II/III stage (19 vs36)	2.065	0.740-5.761	0.166			
Less than vs at least VGPR before ASCT (19 vs 36)	1.646	0.333-2.056	0.683			
Less than vs at least VGPR at three months after ASCT (19 vs 36)	1.646	0.660-4.103	0.285			

t(11;14) group, defined as with t(11;14) and without t(4;14), t(14;16), and del 17p.

Hb, Hemoglobin; BMPC, Bone marrow plasma cells; ISS, International Staging System; LDH, lactate dehydrogenase; VGPR, very good partial remission; ASCT, autologous stem cell transplantation.

**Table 5:** Effect of baseline characteristics and response on OS in the t(11;14) group (n=55).

Variables	Univariate analysis			Multivariate analysis		
	HR	95%CI	p	HR	95%CI	p
Age ≧̸50 vs <50 years (30 vs 25)	2.181	0.484-9.829	0.310	1.206	0.217-6.716	0.831
Hb ≧̸10 vs <10 g/dl (34 vs 21)	0.546	0.106-2.820	0.470			
BMPC ≧̸30 vs <30% (29 vs 26)	0.409	0.079-2.126	0.288	0.821	0.122-5.546	0.840
With vs without CD20 expression (18 vs37)	0.667	0.078-5.709	0.711			
with vs without 1q gain (21 vs 33)	0.314	0.057-1.725	0.183	0.355	0.056-2.255	0.272
Light chain myeloma vs others (20 vs 35)	1.018	0.196-5.280	0.983			
ISS I vs II/III stage (19 vs36)	1.614	0.313-8.334	0.568			
Less than vs at least VGPR before ASCT (19 vs 36)	0.362	0.069-1.881	0.227	0.486	0.084-2.821	0.422
Less than vs at least VGPR at three months after ASCT (19 vs 36)	0.769	0.149-3.978	0.754			

t(11;14) group, defined as with t(11;14) and without t(4;14), t(14;16), and del 17p.

Hb, Hemoglobin; BMPC, Bone marrow plasma cells; ISS, International Staging System; LDH, lactate dehydrogenase; VGPR, very good partial remission; ASCT, autologous stem cell transplantation.

## Discussion

In this multicenter retrospective study, we investigated the clinical manifestation and survival of MM patients with t(11;14) in the era of novel agent in combination with ASCT. The higher proportion of light chain MM, or IgD myeloma for MM patients with t(11;14), are all similar to those in the prior studies. Our study showed patients with t(11;14) had poorer response to induction therapy, but patients in the t(11;14) group had comparable median PFS and OS to the standard risk MM. In addition, the survival tendency was still similar between these two groups regardless of with or without 1q21 gain. Last but not least, we found that both 1q21 gain and bone marrow plasma cell with CD20 expression had no impact on median PFS or OS for t(11;14) patients in multivariate analysis.

In conventional agent era, the early study showed that the outcomes were comparable between MM patients with or without t(11;14). In a transplant ineligible cohort with 336 evaluable patients (1), Fonseca et al. showed that there were no differences in terms of survival and response to treatment between patients with (n=53, 16%) or without t(11;14)(q13;q32) (1). In another transplant eligible cohort from IFM (6), the majority of MM patients received induction therapy with VAD (vincristin, adriamycin, and dexamethasone) and consolidation with ASCT (allogeneic stem cell transplantation for 10 patients). OS for 26 patients (15.5%) with t(11;14)(q13;q32) was longer than patients without this translocation, but the difference between them was marginal (P=0.055). Similar results were observed by other studies as well (3, 7). In general, in conventional agent era, studies showed that outcomes were similar between patients with or without t(11;14), and consolidation with or without ASCT had no impact on survival comparison between them.

However, in novel agent era, the results from some studies cast doubt on the outcomes between patients with or without t(11;14). Lakshman et al. compared PFS and OS between patients with t(11;14) (n=365) and matched controls (n=730). The controls included 132 patients with non-(11;14) abnormality and 598 patients with no chromosomal abnormality. Both PFS and OS of the t(11;14) group were significantly shorter than those of group with no translocation (9). Among patients in this study, the majority received novel agents as induction therapy, but only about 60% received sequential ASCT as consolidation. However, from analysis of ASCT cohort at the Mayo Clinic, it seemed that ASCT could not overcome the adverse impact of t(11;14) (8). In another study from Emory University, among 867 MM patients, 122 patients were detected with t(11;14) by FISH and were compared with 527 with standard risk. All patients received bortezomib, lenalidomide, and dexamethasone as homogeneous induction therapy, but the authors did not mention whether that ASCT had be used as consolidation in their abstract. The results showed that both at least VGPR to induction and PFS for t(11;14) were inferior to those in the standard group (17).

Opposite to the above point of view, several other studies suggested patients with t(11;14) still had the similar outcome compared with patients with standard risk when they received novel agents regimens as induction following ASCT. In the IFM 2009 study (10), they found that the survival of patients with t(11;14) alone did not differ from those with normal karyotype/FISH. In addition, Japanese researchers found that patients with t(11;14) had similar OS to those with normal karyotype/FISH (18). They confirmed further that the additional chromosomal abnormalities from G banding may have an impact on the outcome of patients with t(11:14) (18). More recently, in order to minimize the bias, Neeraj Saini et al. took advantage of a 1:1 propensity score matching technique to compare the outcomes between patients with t(11;14) and with standard risk (11). In this ASCT cohort with 80 patients in each group, they found that there were no significant differences between PFS and OS in the t(11:14) group and those in the control group. Further analyses showed that this was the case for survival comparison between the t(11:14) alone group and the control group. Besides the above analyses, the authors especially emphasized t(11:14) translocation by conventional cytogenetics may have adverse impact on the outcome, and this partially explains the adverse impact of t(11:14) on the outcome in their previous paper (19). In our cohort, the novel agents based induction therapy was used for 90% MM patients, and then all patients received ASCT as consolidation therapy. Our results showed that the patients with t(11;14) alone group have similar outcomes compared with patients with standard risk. The survival tendency for t(11;14) in our study is consistent with the IFM 2009 study and Neeraj Saini et al.’s study.

How do we explain the controversy among the above studies? The limited data from the above studies showed that if novel agents based induction therapy in combination with ASCT was used as treatment for all patients, the outcome of t(11;14) would be similar to a standard risk group. Otherwise, the survival for t(11;14) appeared to be inferior to patients with standard risk. All patients benefited from the novel agents, but it seemed that the outcome of standard risk patients benefited more from novel agents based induction than that of the t(11;14) patients. However, the difference of outcome between patients with t(11;14) and standard risk could be overcome by ASCT. A study from Sweden also confirmed our speculation (12).

A gain of 1q21 is prevalent in 30% to 50% of patients with NDMM (20, 21). The outcome of 1q21 gain in MM remains in dispute. Our previous studies have found 1q21 gain was a statistically independent adverse predictor for PFS (21). However, other studies have failed to conﬁrm this ﬁnding (22, 23). Few researchers have investigated the impact of 1q21 gain on the outcome of MM with t(11;14). Merav Leiba et al. has shown that t(11:14) had high possibility to coexist with 1q21 gain (24). In this study, among patients with t(11;14), 43 were confirmed with 1q21 gain and 6 patients with 1q21 gains. In multivariate analysis, 1q21 gain had no impact on the OS (p=0.257) and it was unreliable for 1q21 gains impact on the OS because of the limited sample size. In our cohort, 1q21 gain was detected in 39.7% of 446 patients and in 38.9% of 54 patients with t(11;14). Therefore, it is necessary to confirm the prognostic value of 1q21 gain for t(11;14) in multivariate analysis. The previous studies showed that bone marrow plasma cell with “CD20 expression (4, 25),” “Age≧̸65 years,” “ISS III stage,” “17p deletion,” and “conventional induction therapy” (26) had been associated with inferior outcome. In our study, besides 1q21 gain and bone marrow plasma cell with CD20 expression, no variables were found to predict median PFS and OS of t(11;14) group in multivariate analysis, which was different from the previous reports (4, 25).

In our study, although patients with t(11;14) had poorer response to induction, no differences were observed in the percentage of patients who achieved at least VGPR in the three groups after ASCT. It was possible for t(11;14) MM to benefit more from ASCT in terms of response compared to the other two groups. Further analysis did not confirm that busulfan containing regimens did better than melphalan containing regimens in terms of at least VGPR after ASCT. Patients with R-ISS-I have similar outcome to patients with R-ISS-II & III, which was attributed to the higher proportion of patients with R- ISS-II among the R-ISS-II & III group (R-ISS-II 58.2% versus R-ISS-III 7.3%). Although no variables can be confirmed to predict the outcome, there was a discrepancy for both PFS and OS of t(11;14) alone. Therefore, more research work should be done to risk-stratify the patients with t(11;14) alone.

There are some shortcomings that should be taken into account when interpreting our results, for instance, heterogeneity in terms of induction, conditioning regimens, and so on. It should be acknowledged that this is a retrospective study and our conclusions have to be tested in future prospective cohort studies.

## Conclusions

With treatment of novel agents induction in combination with ASCT, MM patients with t(11;14) had lower at least VGPR to induction therapy, but had comparable median PFS and OS with standard risk patients. For MM patients with t(11;14), both bone marrow plasma cells with CD20 expression and 1q21 gain had no impact on median PFS or OS.

## Data Availability Statement

The raw data supporting the conclusions of this article will be made available by the authors, without undue reservation.

## Ethics Statement

The studies involving human participants were reviewed and approved by the Ethics Committee of Beijing Chao-Yang Hospital. The patients/participants provided their written informed consent to participate in this study. Written informed consent was obtained from the individual(s) for the publication of any potentially identifiable images or data included in this article.

## Author Contributions

WC, JL, and WF were the principal study investigators for this study and participated in the study design and manuscript revision. WC, JL, and WF should be considered joint corresponding authors. WG, JD, JRL, and HZ participated in data collection, data analysis, and manuscript drafting. WG, JD, JRL, and HZ contributed equally to this work and should be considered joint first authors. ZZ and YJ participated in data collection. GY and GW participated in the execution of statistical analyses. YT, YL, and YW participated in following up with the patients. All authors contributed to the article and approved the submitted version.

## Funding

This project was supported by grant and contract from clinical innovation project of Beijing Hospital Authority [grant XMLX201847]. The funder was not involved in the execution of the research or the preparation of the manuscript.

## Conflict of Interest

The authors declare that the research was conducted in the absence of any commercial or ﬁnancial relationships that could be construed as a potential conﬂict of interest.

## Supplementary Material

Supplementary Table 1 The Conditioning Regimens of the Study Populations at Diagnosis (n=455). t (11;14) group, defined as with t(11;14) and without t(4;14), t(14;16), and del 17p; Standard risk, defined as the absence of del17p, t(4;14), t(14;16), and t(11;14); High risk, defined as the presence of any of del17p, t(4;14), and/or t(14;16). P-value for Fisher’s exact test for categorical variables. BCE, busulfan and cyclophosphamide plus etoposide; TBI, total body irradiation

Supplementary Table 2 The Maintenance of the Study Populations at Diagnosis (n=455). t (11;14) group, defined as with t(11;14) and without t(4;14), t(14;16), and del 17p; Standard risk, defined as the absence of del17p, t(4;14), t(14;16), and t(11;14); High risk, defined as the presence of any of del17p, t(4;14), and/or t(14;16). P-value for Fisher’s exact test for categorical variables. Novel agents, defined as the presence of any of bortezomib, ixazomib, and/or lenalidomide. Conventional agents, defined as the presence of any of thalidomide, and/or interferon.

Supplementary Table 3 Impact of conditioning regimens on response in the t(11;14) group (n=55). Melphalan containing regimens, defined as Melphalan alone, Melphalan plus Bortezomib, and Melphalan plus TBI; Busulfan containing regimens, defined as busulfan and cyclophosphamide plus etoposide, and Busulfan plus Cyclophosphamide. P-value for Fisher’s exact test for categorical variables. VGPR, very good partial remission; ASCT, autologous stem cell transplant.

## Abbreviations

ASCT, Autologous stem cell transplantation, CR, complete response, FISH, fluorescence *in situ* hybridization, IMWG, ISS,, International Myeloma Working Group, international staging system, MR minimal response, multiple myeloma, MM; PR, partial response, PD, progressive disease, PFS, progression free survival, PI, proteasome inhibitors, R-ISS, revised international staging system, OS, overall survival, SD stable disease, sCR, stringent complete response, VGPR, very good partial response, VAD, vincristin, adriamycin, and dexamethasone.
