# Supplementary material for: What Multiple Myeloma With t(11;14) Should Be Classified Into in Novel Agent Era: Standard or Intermediate Risk?
Source: Front Oncol. 2020 Oct 26;10:538126. doi: 10.3389/fonc.2020.538126 (PMC7649769; doi:10.3389/fonc.2020.538126)
Supplement: Supplementary Table 1 — The Conditioning Regimens of the Study Populations at Diagnosis (n=455). t (11;14) group, defined as with t(11;14) and without t(4;14), t(14;16), and del 17p; Standard risk, defined as the absence of del17p, t(4;14), t(14;16), and t(11;14); High risk, defined as the presence of any of del17p, t(4;14), and/or t(14;16). P-value for Fisher’s exact test for categorical variables. BCE, busulfan and cyclophosphamide plus etoposide; TBI, total body irradiation [file Table_1.docx]

**Supplementary TABLE 1**. The Conditioning Regimens of the Study Populations at Diagnosis (n=455)

|  | All patients | t (11;14) group | Standard risk | High risk | P |
| --- | --- | --- | --- | --- | --- |
| Characteristic | （n=455） | 55 | 248 | 152 |  |
| Melphalan alone，n (%) | 224 (49.2） | 30 (54.5） | 126 (50.8) | 68 (44.7) | 0.125 |
| Other regimens，n (%) | 231 (50.8） | 25 (45.5） | 122 (49.2) | 81 (55.3) | 0.125 |
| BCE | 199 (43.7） | 20 (36.4) | 106 (42.7) | 73 (48) | 0.292 |
| Melphalan and Bortezomib | 24 (5.3） | 4 (7.3) | 12 (4.8) | 8 (5.3) | 0.723 |
| Busulfan and Cyclophosphamide | 7 (1.5） | 1 (1.8) | 3 (1.2) | 3 (2.0) |  |
| Melphalan and TBI | 1 (0.22） | 0 | 1 (0.4) | 0 |  |

Note: t (11;14) group, defined as with t(11;14) and without t(4;14), t(14;16), and del 17p; Standard risk, defined as the absence of del17p, t(4;14), t(14;16) and t(11;14); High risk, defined as the presence of any of del17p, t(4;14), and/or t(14;16). P-value for Fisher’s exact test for categorical variables.

Abbreviation: BCE, busulfan and cyclophosphamide plus etoposide; TBI, total body irradiation
