# Supplementary material for: What Multiple Myeloma With t(11;14) Should Be Classified Into in Novel Agent Era: Standard or Intermediate Risk?
Source: Front Oncol. 2020 Oct 26;10:538126. doi: 10.3389/fonc.2020.538126 (PMC7649769; doi:10.3389/fonc.2020.538126)
Supplement: Supplementary Table 2 — The Maintenance of the Study Populations at Diagnosis (n=455). t (11;14) group, defined as with t(11;14) and without t(4;14), t(14;16), and del 17p; Standard risk, defined as the absence of del17p, t(4;14), t(14;16), and t(11;14); High risk, defined as the presence of any of del17p, t(4;14), and/or t(14;16). P-value for Fisher’s exact test for categorical variables. Novel agents, defined as the presence of any of bortezomib, ixazomib, and/or lenalidomide. Conventional agents, defined as the presence of any of thalidomide, and/or interferon. [file Table_2.docx]

**Supplementary TABLE 2**. The Maintenance of the Study Populations at Diagnosis (n=455)

|  | All patients | t (11;14) group | Standard risk | High risk | P |
| --- | --- | --- | --- | --- | --- |
| Characteristic | （n=455） | 55 | 248 | 152 |  |
| Novel agents, n (%) | 97 (21.3) | 14 (25.5) | 51 (20.6) | 32 (21.1) | 0.715 |
| Bortezomib | 56 (12.3) | 9 (16.4) | 31 (12.5) | 16 (10.5) | 0.500 |
| Lenalidomide | 36 (7.9) | 4 (7.3) | 18 (7.3) | 14 (9.2) | 0.775 |
| Bortezomib and Lenalidomide | 3 (0.4) | 0 | 1 (0.4) | 2 (1.3) |  |
| Ixazomib | 2 (0.4) | 1 (1.8) | 1 (0.4) | 0 |  |
| Conventional agents, n (%) | 311 (68.4) | 41 (74.5) | 197 (79.4) | 120 (78.9) | 0.715 |
| Thalidomide | 274 (60.2) | 27 (49.1) | 153 (61.7) | 94 (61.8) | 0.202 |
| Interferon | 22 (4.8) | 5 (9.1) | 16 (6.5) | 1 (0.7) | 0.003 |
| Thalidomide and Interferon | 15 (3.3) | 1(1.8) | 10 (4.0) | 4 (2.6) | 0.695 |
| No, n (%) | 47 (10.3) | 8 (14.5) | 18 (7.3) | 21 (13.8) | 0.053 |

Note: t (11;14) group, defined as with t(11;14) and without t(4;14), t(14;16), and del 17p; Standard risk, defined as the absence of del17p, t(4;14), t(14;16) and t(11;14); High risk, defined as the presence of any of del17p, t(4;14), and/or t(14;16). P-value for Fisher’s exact test for categorical variables. Novel agents, defined as the presence of any of bortezomib, ixazomib, and/or lenalidomide. Conventional agents, defined as the presence of any of thalidomide, and/or interferon.
