# Supplementary material for: What Multiple Myeloma With t(11;14) Should Be Classified Into in Novel Agent Era: Standard or Intermediate Risk?
Source: Front Oncol. 2020 Oct 26;10:538126. doi: 10.3389/fonc.2020.538126 (PMC7649769; doi:10.3389/fonc.2020.538126)
Supplement: Supplementary Table 3 — Impact of conditioning regimens on response in the t(11;14) group (n=55). Melphalan containing regimens, defined as Melphalan alone, Melphalan plus Bortezomib, and Melphalan plus TBI; Busulfan containing regimens, defined as busulfan and cyclophosphamide plus etoposide, and Busulfan plus Cyclophosphamide. P-value for Fisher’s exact test for categorical variables. VGPR, very good partial remission; ASCT, autologous stem cell transplant. [file Table_3.docx]

**Supplementary TABLE 3**. Impact of conditioning regimens on response in the t(11;14) group (n=55)

| The response at three months after ASCT | Melphalan containing regimens  (n=34) | | |  |  | Busulfan containing regimens  (n=21) | | P |
| --- | --- | --- | --- | --- | --- | --- | --- | --- |
| Less then VGPR | | 14 |  | |  | 5 |  | |
| At least VGPR | | 20 |  | |  | 16 | 0.248 | |

Note: Melphalan containing regimens, defined as Melphalan alone, Melphalan plus Bortezomib, and Melphalan plus TBI; Busulfan containing regimens, defined as busulfan and cyclophosphamide plus etoposide, and Busulfan plus Cyclophosphamide. P-value for Fisher’sexact test for categorical variables.

|  |
| --- |

Abbreviations: VGPR, very good partial remission; ASCT, autologous stem cell transplant.
